# Supplementary figures and images for: Poloxamer-188 as a wetting agent for microfluidic resistive pulse sensing measurements of extracellular vesicles
Source: PLoS One. 2024 May 2;19(5):e0295849. doi: 10.1371/journal.pone.0295849 (PMC11065227; doi:10.1371/journal.pone.0295849)

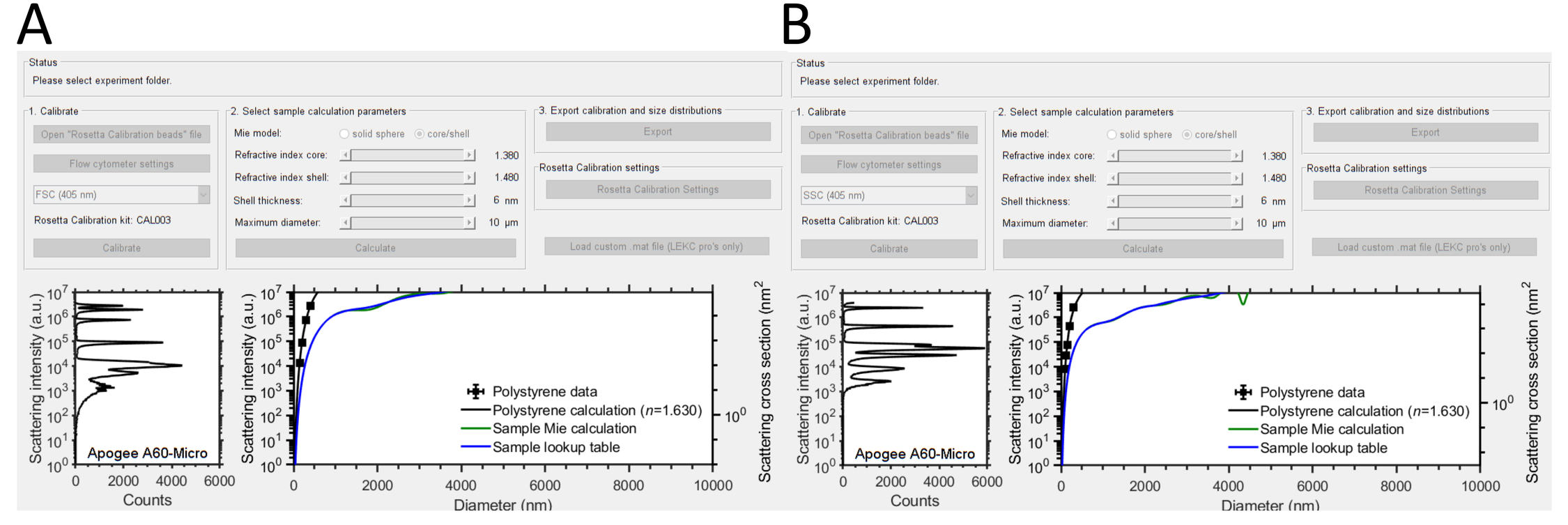

Supplement: S1 Fig — (A) Forward scatter and (B) side scatter calibration of the A60-Micro by Rosetta Calibration. To relate scatter to the size of EVs, we modelled EVs as core-shell particles with a core refractive index of 1.38, a shell refractive index of 1.48, and a shell thickness of 6 nm. (TIF) [file pone.0295849.s001.tif]

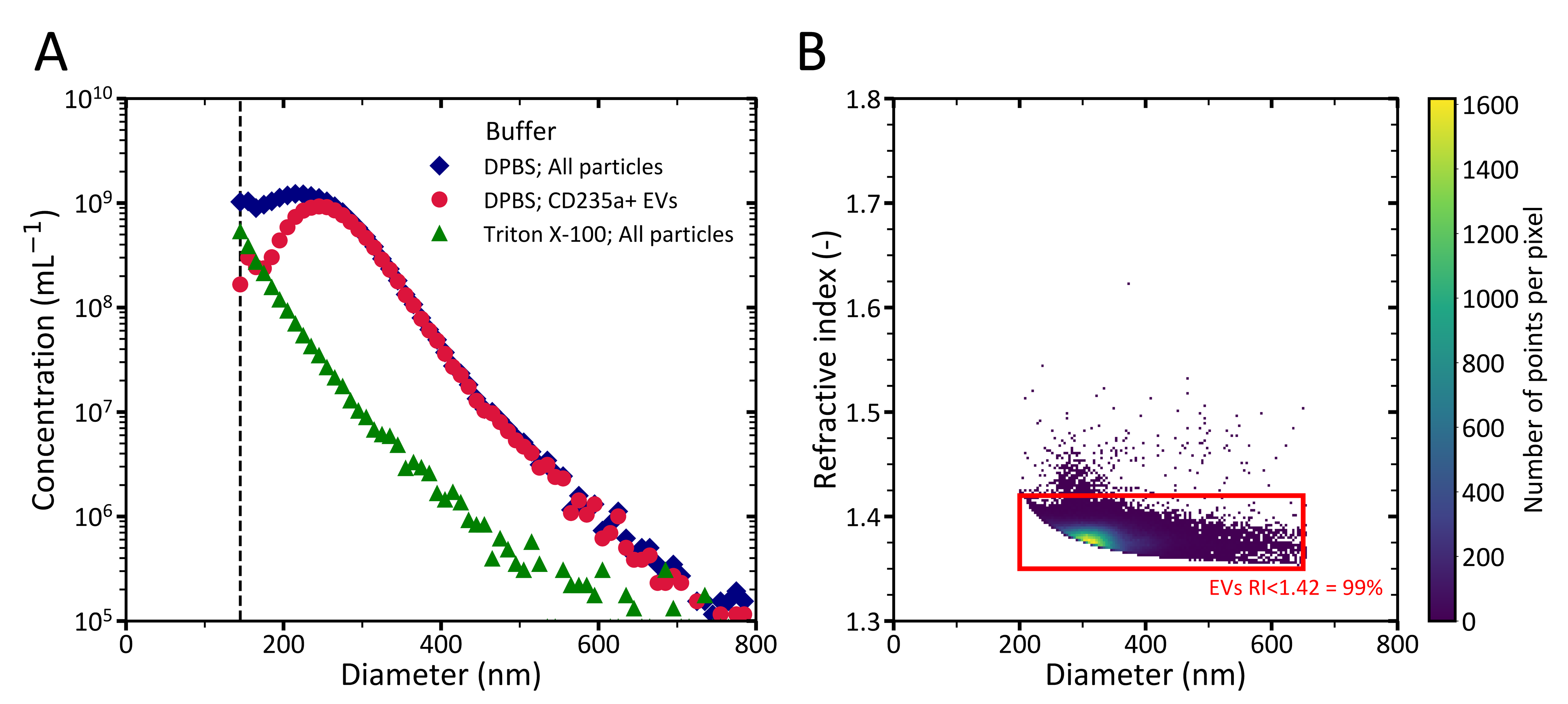

Supplement: S2 Fig — (A) Concentration versus diameter of all particles (diamonds), EVs labeled with CD235a (circles), particles remaining after detergent lysis with 0.10% Triton X-100 in Dulbecco’s phosphate-buffered saline (triangles). To relate scatter to diameter, EVs were modelled as particles with a shell refractive index of 1.48, a shell thickness of 6 nm, and a core refractive index of 1.38. The vertical dashed line marks the trigger threshold for all particles, set at 145 nm. (B) Refractive index versus diameter of particles within the applicable range of the flow cytometry scatter ratio (Flow-SR), which includes 47% of all events. Notably, 99% of these particles show a refractive index below 1.42. (TIF) [file pone.0295849.s002.tif]

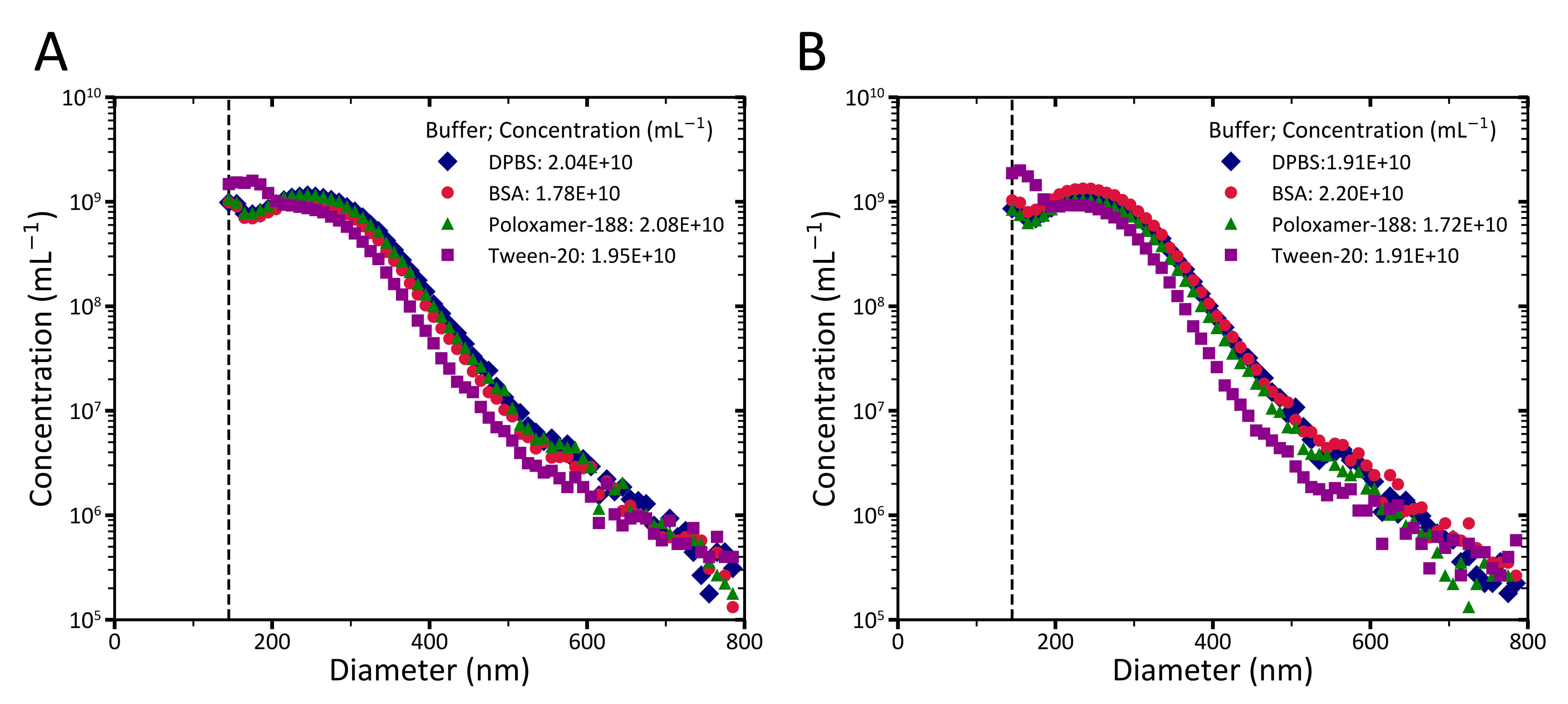

Supplement: S3 Fig — (A, B) Concentration versus diameter of 267- fold diluted EV tests sample in Dulbecco’s phosphate-buffered saline (DPBS; diamond), and DPBS containing 0.10% BSA (w/v; circle), 0.050% Poloxamer-188 (v/v; triangle), and 1.00% Tween-20 (v/v; square). To establish a relationship between scatter and diameter, EVs were modeled as particles with a shell refractive index of 1.48, a shell thickness of 6 nm, and a core refractive index of 1.38. The vertical dashed line shows the trigger threshold of FCM, which is 145 nm. (TIF) [file pone.0295849.s003.tif]

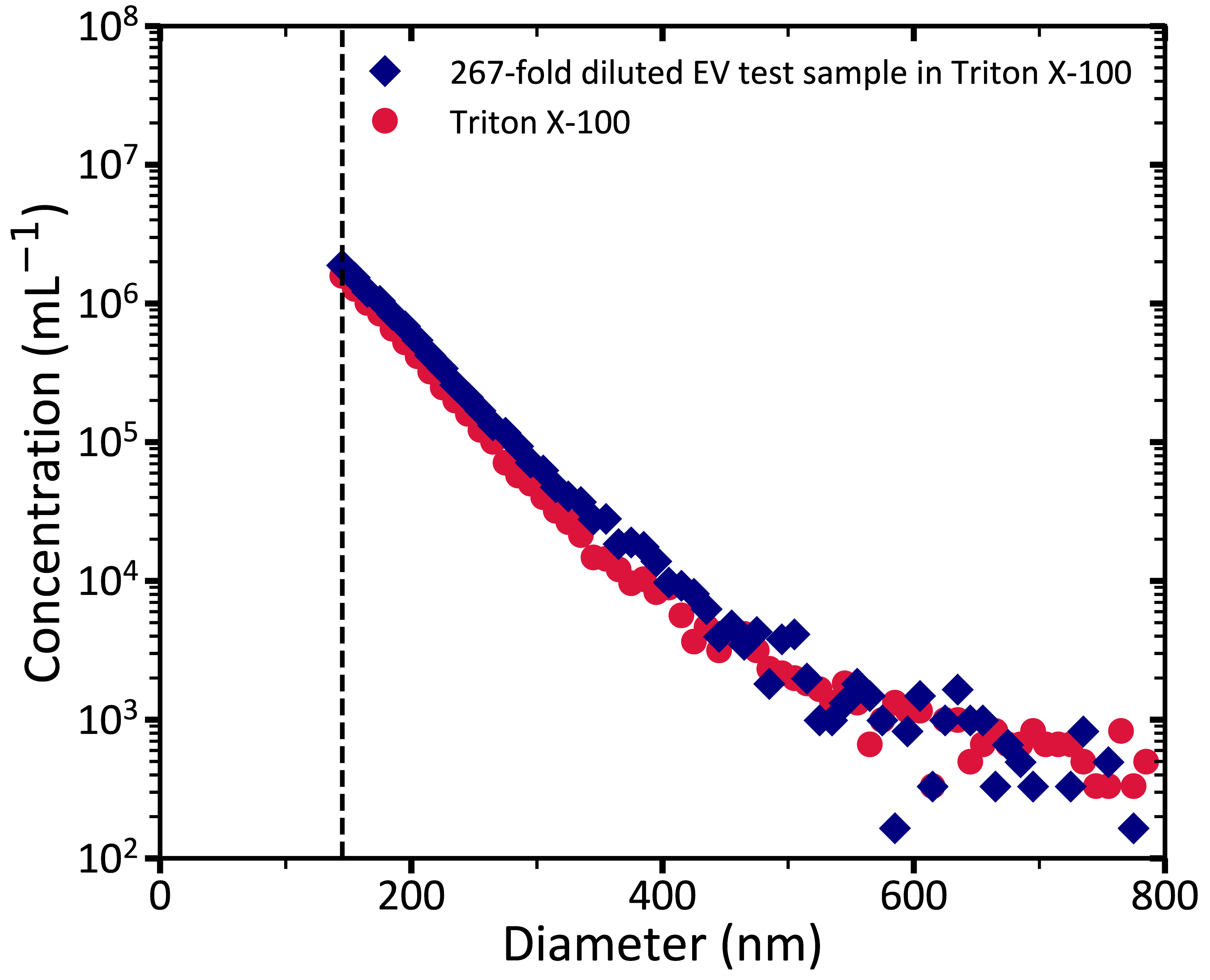

Supplement: S4 Fig — Both size distributions are overlapping and show a steep decrease in concentration with increasing diameter. (TIF) [file pone.0295849.s004.tif]

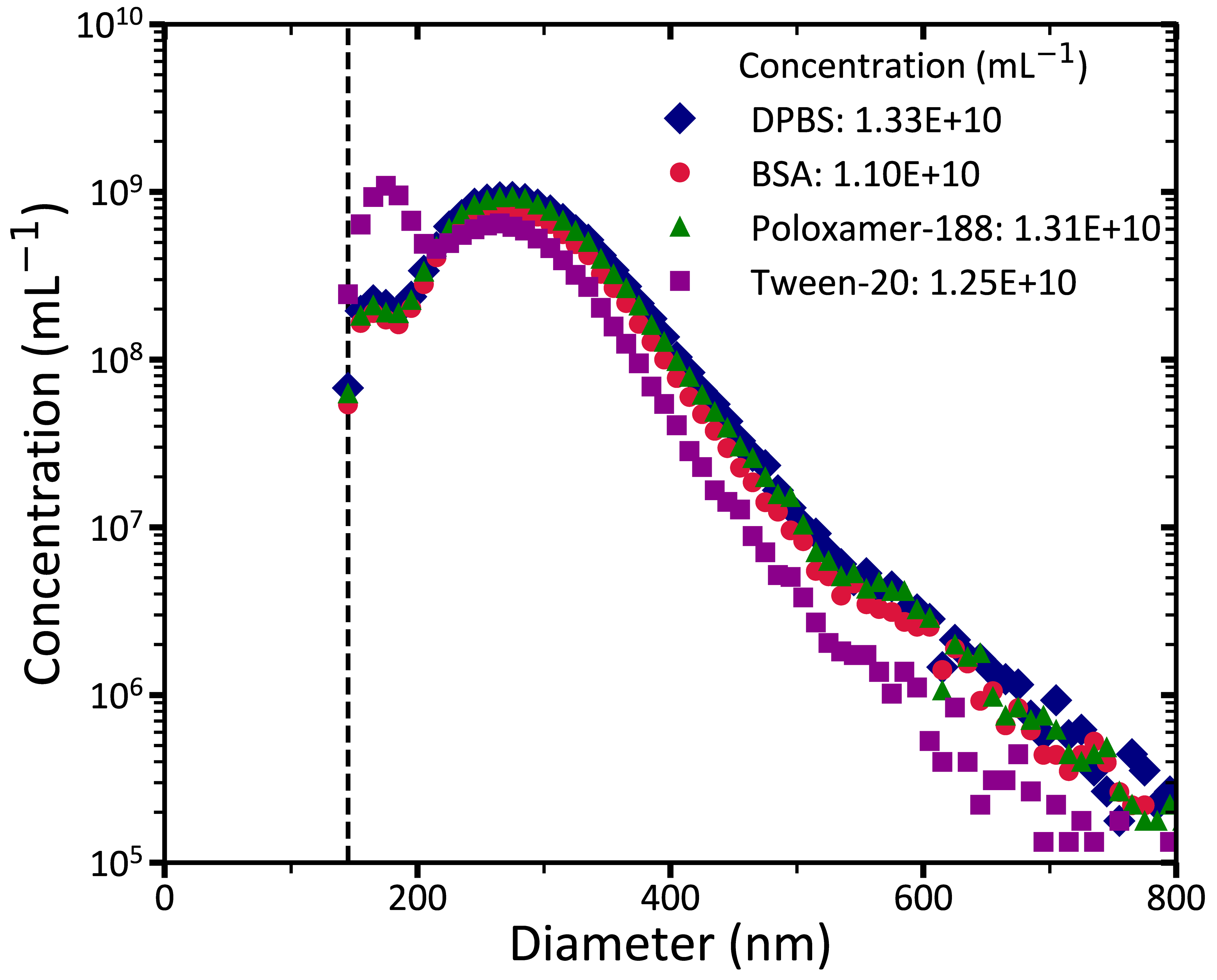

Supplement: S5 Fig — The vertical dashed line represents the trigger threshold of FCM, which is 145 nm. Diamonds, circles, triangles, and squares represent the particle size distribution of CD235a+ EVs in Dulbecco’s phosphate-buffered saline (DPBS) or DPBS containing 0.10% BSA (w/v), 0.050% Poloxamer-188 (v/v), or 1.00% Tween-20 (v/v), respectively. To relate scatter to diameter, EVs were modelled as particles with a core refractive index of 1.38, a shell refractive index of 1.48 and a shell thickness of 6 nm. (TIF) [file pone.0295849.s005.tif]
