# Supplementary material for: Poloxamer-188 as a wetting agent for microfluidic resistive pulse sensing measurements of extracellular vesicles
Source: PLoS One. 2024 May 2;19(5):e0295849. doi: 10.1371/journal.pone.0295849 (PMC11065227; doi:10.1371/journal.pone.0295849)
Supplement: S2 File — (DOCX) [file pone.0295849.s007.docx]

Supporting information 2: MIFlowCyt-EV of “Poloxamer-188 as a wetting agent for microfluidic resistive pulse sensing measurements of extracellular vesicles”

Mona Shahsavari1,2,3^,4,5 ⃰^, Rienk Nieuwland1,2^,5,6^, Ton G. van Leeuwen1^,3,4,5^, Edwin van der Pol1,2,3^,4,5^

1. Amsterdam Vesicle Center, Amsterdam UMC, University of Amsterdam, Amsterdam, the Netherlands
2. Laboratory of Experimental Clinical Chemistry, Amsterdam UMC, University of Amsterdam, Amsterdam, the Netherlands
3. Biomedical Engineering and Physics, Amsterdam UMC, University of Amsterdam, Amsterdam, the Netherlands
4. Amsterdam Cardiovascular Sciences, Atherosclerosis and Ischemic Syndromes, Amsterdam, The Netherlands
5. Cancer Center Amsterdam, Imaging and Biomarkers, Amsterdam, The Netherlands
6. Amsterdam Cardiovascular Sciences, Microcirculation, Amsterdam, the Netherlands

* [m.shahsavari@amsterdamumc.nl](mailto:m.shahsavari@amsterdamumc.nl) (MS)

# Flow cytometry

## Experimental design

The aim of flow cytometry (A60-Micro, Apogee Flow Systems, Hemel Hempstead, UK) experiments was to investigate the impact of three different wetting agents (0.1% bovine serum albumin (BSA; w/v), 0.05% Poloxamer-188 (v/v) or 1% Tween-20 (v/v) in dulbecco's phosphate buffered saline (DPBS)) on the concentration and size distribution of extracellular vesicles (EVs) in an outdated erythrocyte blood bank concentrated sample (EV test sample). The concentration and size distribution of both unstained and CD235+ EVs in the diluted EV test sample in DPBS contained BSA, Poloxamer-188 and Tween-20, were compared to those of the diluted EV test sample in DPBS alone as a positive control. To lyse EVs, the solution of 0.1% Triton X-100 was used as a negative control [1]. Our hypothesis was that the concentration and size distribution of EVs in EV test sample, when diluted in the solutions of 0.1% BSA (w/v) and 0.05% Poloxamer-188 (v/v) would resemble those of the diluted EVs in DPBS.

All samples were measured using an autosampler, which facilitates subsequent measurements of samples in a 96-well plate. For this study, samples were measured in one day. A buffer-only control was measured as well as antibody in buffer controls and isotype controls corresponding to the labels in the well plate. Flow rate and scatter calibrations were performed daily. Fluorescence calibration was performed seven months before the measurement day. To automatically determine optimal sample dilutions, apply calibrations, determine and apply gates, generate reports with scatter plots and generate data summaries, we used custom-build software (MATLAB R2020b, Mathworks, Natick, MA, USA).

## Sample dilutions

To prevent swarm detection [2], the EV test sample was diluted 24-fold in DPBS, followed by an additional 11.125-fold dilution in DPBS in the presence of the desired wetting agents, either after staining or without staining. This dilution strategy resulted in count rates ≤ 5.0∙10^3^ events per second for all measurements, which is below the threshold of swarm detection for our flow cytometer and this sample type [3].

## EV staining

EVs were stained with CD235a- phycoerythrin (PE). Prior to staining, antibodies were diluted in DPBS and centrifuged at 18,890 g for 5 min to remove aggregates. Table 1 shows an overview of the used reagent and antibody concentrations during staining. To stain, 20 μL of pre-diluted EV test sample was incubated with 2.5 μL of antibodies or isotype controls and kept in the dark for 2 h at room temperature. To decrease background fluorescence caused by unbound reagents, the post-staining samples were diluted 11.125-fold in 200 μL of DPBS or DPBS contained 0.1% BSA (w/v), 0.05% Poloxamer-188 (v/v), 0.1% Triton X-100, or 1% Tween-20 (v/v).

## Buffer-only control

DPBS and DPBS containing BSA, Poloxamer, Triton X-100, and Tween-20 were measured using the same flow cytometer and acquisition settings as the samples. The mean count rates obtained were as follows: DPBS – 25.6 events per second, 0.1% BSA (w/v) – 14.1 events per second, 0.05% Poloxamer-188 (v/v) – 11.7 events per second, 0.1% Triton X-100 - 445.3 events per second, and 1% Tween-20 - 192.3 events per second. These count rates are substantially lower than the average count rate obtained for the EV test sample, which was 3615.9 events per second.

## Buffer with reagents control

The 96-wellplate contained buffers with the reagent control (Table 1), which were measured with the same flow cytometer and acquisition settings as the samples. The mean count rate for the different buffers with CD235 control were as follows: 75.1 events per second for DPBS, 78.9 events per second for 0.1% BSA (w/v), 56 events per second for 0.05% Poloxamer-188 (v/v), 449.6 events per second for 0.1% Triton X-100 (v/v), and 392 events per second for 1% Tween-20 (v/v). These count rates fell within the range observed for the buffer-only control.

## Unstained samples

Unstained sample diluted in different buffers were measured with the same flow cytometer and acquisition settings as the stained samples. The mean count rate for the diluted EV test sample in different buffers were as follows: 3615.9 events per second for DPBS, 3770.2 events per second for 0.1% BSA (w/v), 3715.12 events per second for 0.05% Poloxamer-188 (v/v), 557 events per second for 0.1% Triton X-100 (v/v), and 4091.8 events per second for 1% Tween-20 (v/v). These count rates were in the same range as those of the stained samples.

## Isotype controls

Table 1 shows an overview of the used isotype control. For 4 EV test control samples diluted in DPBS, 0.1% BSA (w/v), 0.05% Poloxamer-188 (v/v), and 1% Tween-20 (v/v), we obtained an average of 235.5 IgG1-PE+ events with a diameter ≤1,000 nm per measurement. For comparison, on average of 281946.7 CD235-PE+, events with a diameter ≤1,000 nm were obtained in the experiments using the diluted EV test sample.

## Trigger channel and threshold

Based on the buffer-only control (25.6 events s^-1^), the acquisition software was set up to trigger at 24 arbitrary units (a.u.) side scattering (SSC), which is equivalent to an SSC cross section of 8 nm^2^ (Rosetta Calibration, v1.24, Exometry, Amsterdam, The Netherlands).

## Flow rate quantification

Each measurement day the flow rate was validated internally (Apogee calibration beads, Apogee Flow Systems, Hemel Hempstead, UK). As the A60-Micro is equipped with a syringe pump with volumetric control, we assumed a flow rate of 3.01 μL/min for all measurements.

## Fluorescence calibration

Calibration of the fluorescence detector from arbitrary units (a.u.) to molecules of equivalent soluble fluorochrome (MESF) was accomplished using SPHERO Easy Calibration Fluorescent Particles (AK01, Spherotech Inc., Irma Lee Circle, IL, USA).

Calibration of the PE detector was performed seven months before these experiments. We added fluorescent intensities in MESF to the flow cytometry data files by custom-build software (MATLAB R2020b) using the following equation:

| $\text{I(}\text{MESF}\text{)}= {10}^{a.\log_{10} I\left( a.u. \right)+b}$ | (1) |
| --- | --- |

where I is the fluorescence intensity, and a and b are the slope and the intercept of the linear fits, respectively, see Table 2. At the measurement day, the fluorescence calibration has been corrected for daily deviations using fluorescent beads.

## Light scatter calibration

We used Rosetta Calibration to relate scatter measured by forward scattering (FSC) or SSC to the scattering cross section and diameter of EVs. Fig 1 shows print screens of the scatter calibrations. We modelled EVs as core-shell particles with a core refractive index of 1.38, a shell refractive index of 1.48, and a shell thickness of 6 nm. For each measurement, we added the FSC and SSC scattering cross sections and EV diameters to the flow cytometry datafiles by custom-build software (MATLAB R2020b).

## MIFlowCyt checklist

The MIFlowCyt checklist is added to Table 3.

## EV number concentration

The concentrations reported in the manuscript describe the number of particles (1) that exceeded the SSC threshold, corresponding to a side scattering cross section of 8 nm^2^, (2) that were collected during time intervals, for which the count rate did not deviated more than 750 events/s of the median count rate, (3) with a diameter <1,000 nm as measured by SSC after light scatter calibration (section 1.11) or (4) are positive for PE (lower boundary of the fluorescent gate was 118 MESF for CD235a-PE), per mL of EV test sample.

## Data sharing

Data is available via: https://doi.org/10.6084/m9.figshare.23260886.

# References

1. Osteikoetxea X, Sódar B, Németh A, Szabó-Taylor K, Pálóczi K, Vukman K V., et al. Differential detergent sensitivity of extracellular vesicle subpopulations. Org Biomol Chem. 2015;13: 9775–9782. doi:10.1039/c5ob01451d

2. Van Der Pol E, Van Gemert MJC, Sturk A, Nieuwland R, Van Leeuwen TG. Single vs. swarm detection of microparticles and exosomes by flow cytometry. Journal of Thrombosis and Haemostasis. 2012;10: 919–930. doi:10.1111/j.1538-7836.2012.04683.x

3. Buntsma NC, Shahsavari M, Gąsecka A, Nieuwland R, van Leeuwen TG, van der Pol E. Preventing swarm detection in extracellular vesicle flow cytometry: a clinically applicable procedure. Res Pract Thromb Haemost. 2023;7: 100171. doi:10.1016/j.rpth.2023.100171


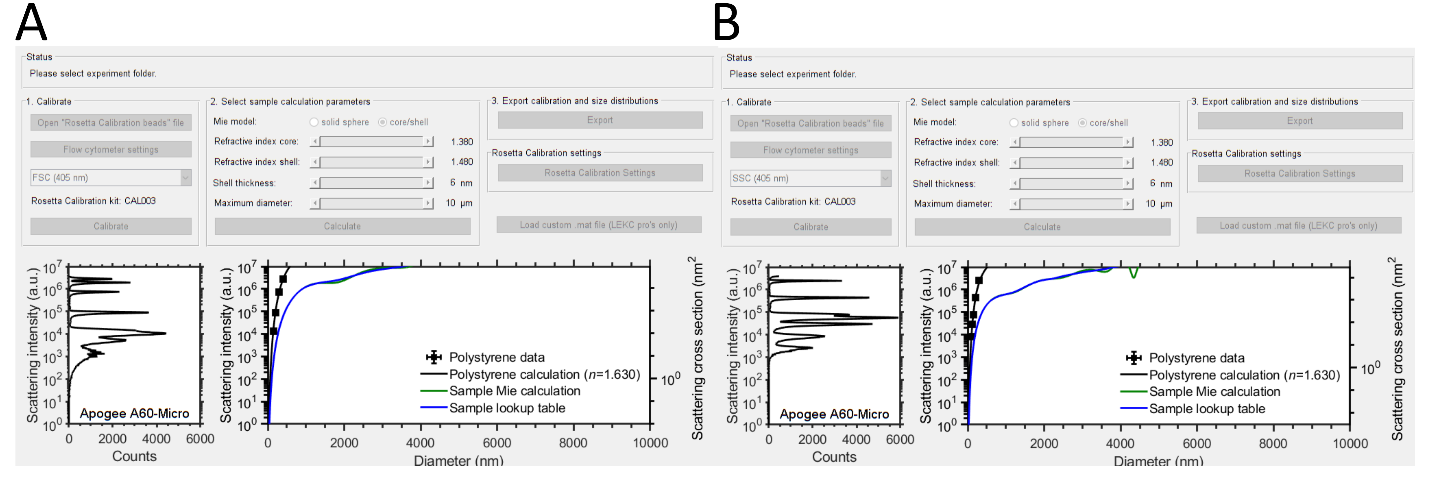


**Fig 1. Scatter calibrations.** (A) Forward scatter and (B) side scatter calibration of the A60-Micro by Rosetta Calibration. To relate scatter to the diameter of EVs, we modelled EVs as core-shell particles with a core refractive index of 1.38, a shell refractive index of 1.48, and a shell thickness of 6 nm.

**Table 1. Overview of staining reagents.**  Characteristics being measured, analyte, analyte detector, reporter, isotype, clone, concentration, manufacturer, catalog number and lot number of used staining reagents. The antibody concentration during measurements was 11.125-fold lower than the antibody concentration during staining.

| **Characteristic**  **measured** | **Analyte** | **Analyte detector** | **Reporter** | **Isotype** | **Clone** | **Concentration during staining (µg mL^-1^)** | **Manufacturer** | **Catalog number** | **Lot number** |
| --- | --- | --- | --- | --- | --- | --- | --- | --- | --- |
| Glycophorin-A | CD235a | Anti-human CD235a  antibody | PE | IgG1 | JC159 | 25 | Dako Agilent | R7078 | 20079786 |
| Affinity for Fc receptor | Fc receptor | IgG1 | PE | n.a. | X40 | 25 | BD Bioscience | 345816 | 9309643 |

IgG: immunoglobulin G; PE: phycoerythrin.

**Table 2. Overview of fluorescence calibrations.**

|  | Calibration date | Slope | Intercept | R^2^ |
| --- | --- | --- | --- | --- |
| PE | 2021-07-15 | 1.0118 | -1.4839 | 0.9995 |

PE: phycoerythrin.

**Table 3. MIFlowCyt checklist.**

| **Requirement** | **Please Include Requested Information** |
| --- | --- |
| - 1. Purpose | To investigate the potential impact of three wetting agents, BSA, Poloxamer-188, and Tween-20 on extracellular vesicles (EVs). A cell-free outdated erythrocyte concentrate sample was used as the EV test sample. The stained and unstained EV test sample was diluted in either filtered DPBS (positive control), or DPBS containing 0.1% BSA (w/v), 0.05% Poloxamer-188 (v/v), 0.1% Triton X-100 (v/v) (negative control), or 1% Tween-20 (v/v). The EV test samples were then analysed using flow cytometry (FCM) to measure their concentration and size distribution. |
| 1.2. Keywords | Poloxamer-188, Tween-20, wetting agents, extracellular vesicles |
| 1.3. Experiment variables | Diluents |
| 1.4. Organization name and address | Amsterdam University Medical Centers  Location Academic Medical Centre  Meibergdreef 9  1105 AZ Amsterdam  The Netherlands |
| 1.5. Primary contact name and email address | Mona Shahsavari, m.shahsavari@amsterdamumc.nl |
| 1.6. Date or time period of experiment | 24/02/2022 |
| 1.7. Conclusions | The concentration and size distribution of unstained and CD235+ EVs are not affected in the presence of 0.1% BSA (w/v) and 0.05% Poloxamer-188 (v/v). |
| 1.8. Quality control measures | All samples were measured using an autosampler, which facilitates subsequent measurements of samples in a 96-well plate. Each well plate contained buffer-only controls (section S1.4), antibody in buffer controls (section S1.5), unstained controls (section S1.6) and isotype controls (section S1.7). The flow rate is validated daily. Fluorescence detectors were calibrated (section S1.10) with SPHERO Easy Calibration Fluorescent Particles (AK01, Spherotech Inc., Irma Lee Circle, IL, USA). FSC and SSC were calibrated with Rosetta Calibration (v1.24, section S1.11). |
| 1.9 Other relevant experiment information | Samples were measured in one day. |
| 2.1.1.1. Sample description | The EV test sample analysed in this study is a cell-free erythrocyte blood bank concentrate sample. |
| 2.1.1.2. Biological sample source description | To remove cells, a 6 weeks old erythrocyte blood bank concentrate sample was centrifuged twice at 2,500g, 15 minutes, 20˚C, with no brake using a Rotina 46RS centrifuge (Hettich Zentrifugen, Tuttlingen, Germany). For freeze-storage, aliquots of 500 µL sample were transferred to 1.5 mL micro tubes (Sarstedt AG & Co., Germany), snap-frozen in liquid nitrogen and stored in -80˚C. Before staining, samples were thawed for 2 minute at 37°C. |
| 2.1.1.3. Biological sample source organism description | Human |
| 2.2 Sample characteristics | Outdated erythrocyte blood bank concentrate sample is anticipated to mostly contain EVs. However, it also contains lipoproteins and proteins in addition to EVs. |
| 2.3. Sample treatment description | The erythrocyte blood bank concentrate sample was kept in the fridge for 6 weeks. EV test sample was prepared by double centrifugation and samples were stained. Please see section S1.3 for staining procedure. |
| 2.4. Fluorescence reagent(s) description | Please see Table 1. |
| 3.1. Instrument manufacturer | Apogee, Hemel Hempstead, UK |
| 3.2. Instrument model | A60-Micro |
| 3.3. Instrument configuration and settings | Samples were analysed for 2 minutes at a flow rate of 3.01 μL/min on an A60-Micro, equipped with a 405 nm laser (100 mW), 488 nm laser (150 mW) and 638 nm laser (150 mW). The trigger threshold was set at SSC 24 a.u. corresponding to an SSC cross section of 8 nm^2^ (Rosetta Calibration).  For FSC and SSC, the PMT voltages were 470 V and 375 V, respectively. For all detectors, the peak height was analysed. PE signals were collected with the 488-Orange (Peak) detector (575/30 nm band pass filter, PMT voltage 450 V). |
| 4.1. List-mode data files | Data is available via: https://doi.org/10.6084/m9.figshare.23260886. |
| 4.2. Compensation description | No compensation was required because no fluorophore combinations were used that have overlapping emission spectra. |
| 4.3. Data transformation details | Fluorescence detectors were calibrated (section S1.10) with SPHERO Easy Calibration Fluorescent Particles (AK01, Spherotech Inc., Irma Lee Circle, IL, USA).  FSC and SSC were calibrated with Rosetta Calibration (v1.24, section S1.11). The concentrations reported in the manuscript describe the number of particles that fulfil the gating criteria per mL. |
| 4.4.1. Gate description | To automatically apply gates, generate pdf reports with scatter plots, and summarize the data in a table, custom-build software (MATLAB R2020b) was used. Please find below a description of the gates. First, events that were collected during seconds for which the count rate deviated less than 750 events/seconds from the median count rate were included. Second, events with a diameter <1,000 nm as measured by SSC after light scatter calibration (section S1.11) were included. Third, events positive for PE were included. |
| 4.4.2. Gate statistics | The number of positive events was corrected for flow rate, measurement time and dilutions performed during sample preparation. |
| 4.4.3. Gate boundaries | The lower boundary of the fluorescent gate was automatically determined (MATLAB R2020b), resulting in 118 MESF for CD235a-PE. |

DPBS: dulbecco's phosphate buffered saline; BSA: bovine serum albumin; a.u.: arbitrary units; FSC: forward scattering; SSC: side scattering.
