# Supplementary material for: Poloxamer-188 as a wetting agent for microfluidic resistive pulse sensing measurements of extracellular vesicles
Source: PLoS One. 2024 May 2;19(5):e0295849. doi: 10.1371/journal.pone.0295849 (PMC11065227; doi:10.1371/journal.pone.0295849)
Supplement: S3 File — (DOCX) [file pone.0295849.s008.docx]

Supplemental information 3: Supporting data

Mona Shahsavari1,2,3^,4,5 ⃰^, Rienk Nieuwland1,2^,5,6^, Ton G. van Leeuwen1^,3,4,5^, Edwin van der Pol1,2,3^,4,5^

1. Amsterdam Vesicle Center, Amsterdam UMC, University of Amsterdam, Amsterdam, the Netherlands
2. Laboratory of Experimental Clinical Chemistry, Amsterdam UMC, University of Amsterdam, Amsterdam, the Netherlands
3. Biomedical Engineering and Physics, Amsterdam UMC, University of Amsterdam, Amsterdam, the Netherlands
4. Amsterdam Cardiovascular Sciences, Atherosclerosis and Ischemic Syndromes, Amsterdam, The Netherlands
5. Cancer Center Amsterdam, Imaging and Biomarkers, Amsterdam, The Netherlands
6. Amsterdam Cardiovascular Sciences, Microcirculation, Amsterdam, the Netherlands

* [m.shahsavari@amsterdamumc.nl](mailto:m.shahsavari@amsterdamumc.nl) (MS)

# Replication of the EV test sample characterization

To increase reliability of our findings, Fig 1 shows a replicate experiment of Fig 2 of the manuscript. In Fig 1A, we observe that 64% of the particles >145 nm are labeled with CD235a-phycoerythrin (PE) and 88% of the particles undergo lysis upon the addition of 0.10% Triton X-100 to the EV test sample. Fig 1B shows that 99% of the particles falling within the applicable range of the flow cytometry scatter ratio (Flow-SR) have a refractive index <1.42. The abovementioned results are in good agreement with the outcomes presented in Fig 2 of the manuscript.





**Fig 1. Flow cytometry analysis of EV test Sample.** (A) Concentration versus diameter of all particles (diamonds), EVs labeled with CD235a (circles), particles remaining after detergent lysis with 0.10% Triton X-100 in Dulbecco's phosphate-buffered saline (triangles). To relate scatter to diameter, EVs were modelled as particles with a shell refractive index of 1.48, a shell thickness of 6 nm, and a core refractive index of 1.38. The vertical dashed line marks the trigger threshold for all particles, set at 145 nm. (B) Refractive index versus diameter of particles within the applicable range of the flow cytometry scatter ratio (Flow-SR), which includes 47% of all events. Notably, 99% of these particles show a refractive index below 1.42.

# Replication of the effect of wetting agents on EVs

To enhance the credibility of our findings regarding the influence of wetting agents on EVs, as presented in Fig 3 of the manuscript, we conducted two independent replicate experiments. Fig 2A and Fig 2B show the results of these replicated measurements, demonstrating the measured concentration and size distribution of EVs in the diluted EV test sample in Dulbecco’s phosphate-buffered saline (DPBS), DPBS containing 0.10% bovine serum albumin (BSA; w/v), 0.05% Poloxamer-188 (v/v), and 1.00% Tween-20 (v/v). It is shown that the concentration and size distribution of EVs in the presence of Poloxamer-188 closely resemble those of the controls (EV test sample diluted in DPBS and BSA). However, the size distribution of EVs in the presence of Tween-20 deviates from that of the controls. In Fig 2A and 2B, we observe a 29% and 41% increase in the concentration of EVs smaller than 235 nm in the presence of Tween-20, respectively, while the concentration of EVs larger than 235 nm decreases by 40% and 32%. These replications align with the findings reported in Fig 3 of the manuscript.





***Fig 2.* Two independent measurements of the extracellular vesicle (EV) test sample with flow cytometry (FCM).** (A, B) Concentration versus diameter of 267- fold diluted EV tests sample in Dulbecco's phosphate-buffered saline (DPBS; diamond), and DPBS containing 0.10% BSA (w/v; circle), 0.050% Poloxamer-188 (v/v; triangle), and 1.00% Tween-20 (v/v; square). To establish a relationship between scatter and diameter, EVs were modeled as particles with a shell refractive index of 1.48, a shell thickness of 6 nm, and a core refractive index of 1.38. The vertical dashed line shows the trigger threshold of FCM, which is 145 nm.

# Size distribution of particles in Triton X-100

We examined the size distribution of particles in the (267-fold diluted) EV test sample during the flow cytometry measurements in the presence of 0.10% Triton X-100, as shown in Fig 3. This distribution closely resembles the size distribution of particles found in a pure Triton X-100 solution. This observation verifies that the majority of the particles in the EV test sample that remain after detergent lysis are particles in Triton X-100. These particles are most likely micelles.


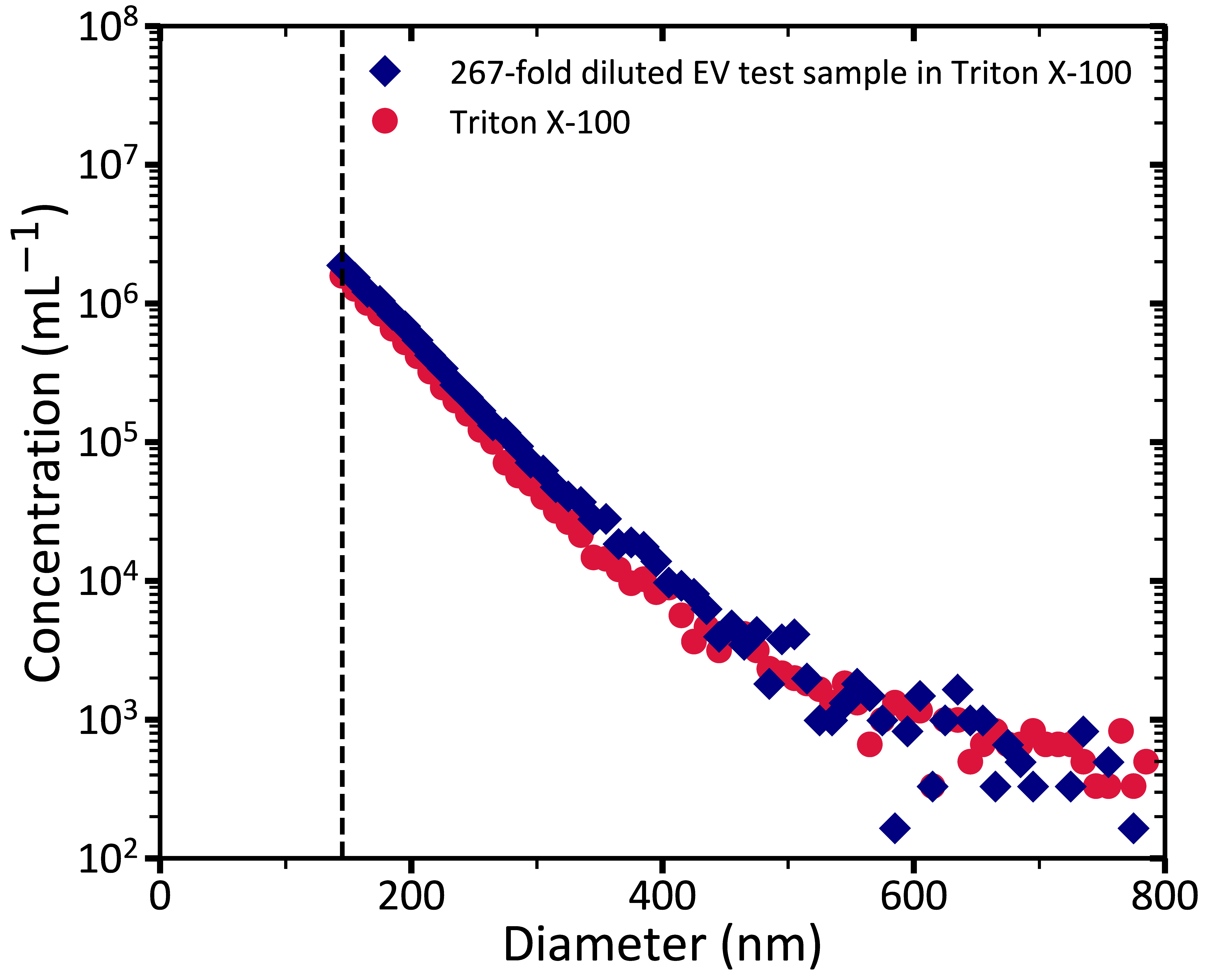


**Fig 3. Concentration of particles in a lysed extracellular vesicle (EV) test sample (diamonds) and in Triton X-100 (circles) versus diameter measured with flow cytometry (FCM)**. Both size distributions are overlapping and show a steep decrease in concentration with increasing diameter.

# Effect of wetting agents on stained extracellular vesicles

Fig 4 shows the concentration and size distribution of CD235a+ EVs in the EV test sample diluted in various solutions: DPBS and DPBS containing 0.10% BSA (w/v), 0.05% Poloxamer-188 (v/v), and 1.00% Tween-20 (v/v). The total concentration of CD235a+ EVs are virtually unaffected by the wetting agents. The size distribution of stained EVs in the presence of BSA and Poloxamer-188 are similar to the size distribution in DPBS. However, when Tween-20 is present, the concentration of CD235a+ EVs< 265 nm increases by 32%, while the concentration of CD235a+ EVs ≥265 nm decreases by 45%. These findings are in agreement with the results obtained for the effect of wetting agents on unstained EVs, as shown in Fig 3 of the manuscript.


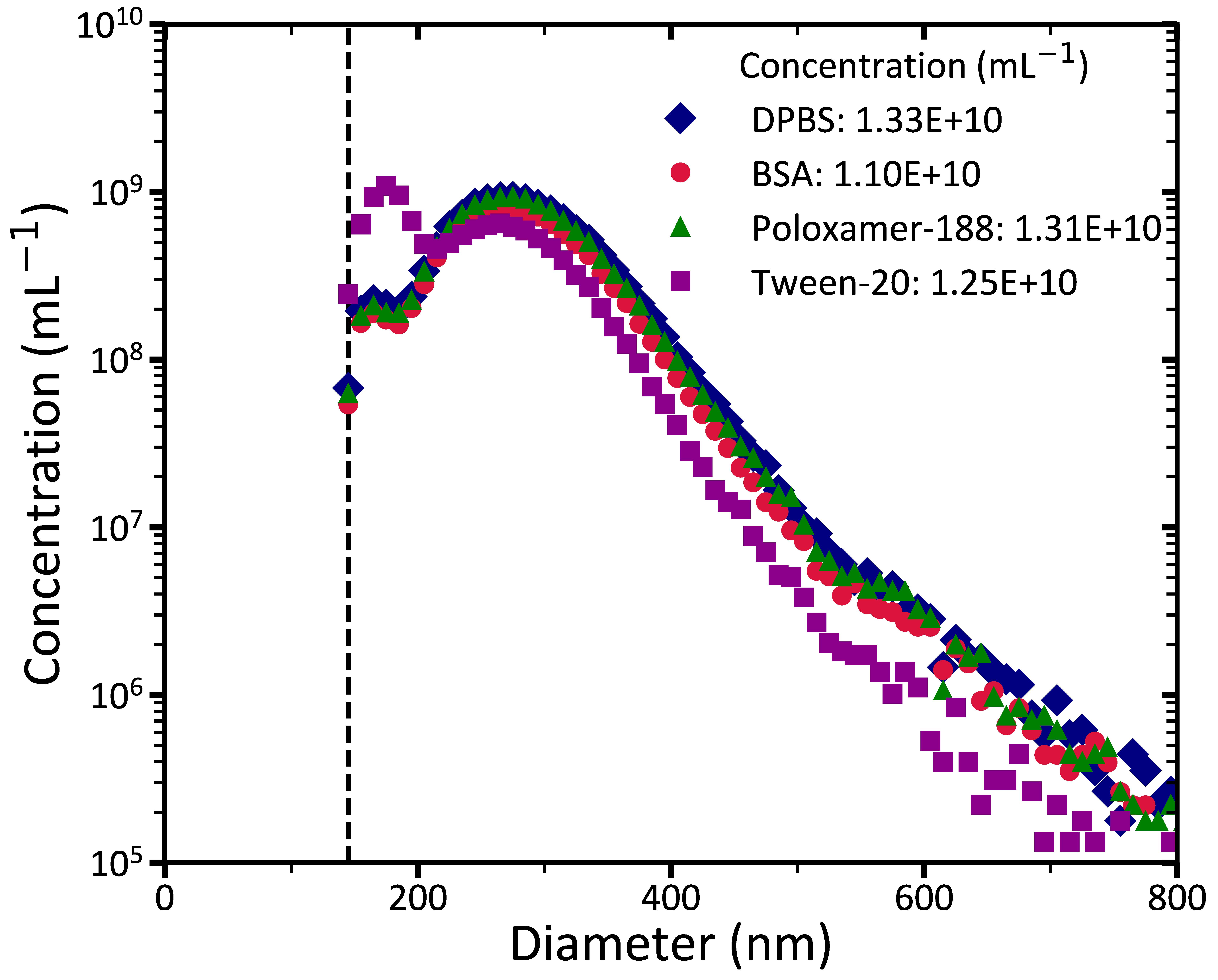


**Fig 4. Concentration versus the diameter of particles in the extracellular vesicle (EV) test sample measured with flow cytometry (FCM).** The vertical dashed line represents the trigger threshold of FCM, which is 145 nm. Diamonds, circles, triangles, and squares represent the particle size distribution of CD235a+ EVs in Dulbecco's phosphate-buffered saline (DPBS) or DPBS containing 0.10% BSA (w/v), 0.050% Poloxamer-188 (v/v), or 1.00% Tween-20 (v/v), respectively. To relate scatter to diameter, EVs were modelled as particles with a core refractive index of 1.38, a shell refractive index of 1.48 and a shell thickness of 6 nm.
