# Supplementary material for: Poloxamer-188 as a wetting agent for microfluidic resistive pulse sensing measurements of extracellular vesicles
Source: PLoS One. 2024 May 2;19(5):e0295849. doi: 10.1371/journal.pone.0295849 (PMC11065227; doi:10.1371/journal.pone.0295849)
Supplement: S1 Data — (ZIP) [file pone.0295849.s009.zip › supporting information/A1 C20161751 d.d. 6 september 2016 (2).pdf]

Van: mecamc
Verzonden: dinsdag 6 september 2016 16:45
Aan: T. van der Poll
Onderwerp: MEC 2015_074

Onderstaande brief wordt u uitsluitend per email gestuurd.

Amsterdam, 6 september 2016
ons kenmerk: 2015_074#C20161751
betreft:
NL52925.018.15
Blood sampling for CEMM experiments

Medisch Ethische Toetsingscommissie
E2-172
telefoon: 020 56 67389

Geachte heer Van der Poll,

Per email d.d. 17 augustus 2016 ontvingen wij van Duncan Kranendonk het herziene protocol betreffende bovengenoemde studie. Dit is besproken in de vergadering van onze commissie van 1 september jongstleden. Daarbij kwam het volgende aan de orde:

In onze brief van 14 april 2015 hebben wij al aangegeven dat het project onder voorwaarden niet WMO-plichtig is.
De commissie is van oordeel dat het protocol en de proefpersoneninformatie nu dusdanig zijn opgezet dat aan de voorwaarden voor niet WMO-plichtigheid voor dit project is voldaan.

Wij delen u gaarne mee deze documenten ter kennisgeving te hebben aangenomen.

Betrokken documenten:
A1 aanbiedingsbrief d.d. 16 maart 2015
A1 aanbiedingsemail d.d. 19 maart 2015
A1 aanbiedingsemail d.d. 20 januari 2016
A1 aanbiedingsemail d.d. 31 mei 2016
A1 aanbiedingsemail d.d. 1 juni 2016
A1 aanbiedingsemail d.d. 17 augustus 2016
B2 AMC appendix getekend d.d. 25 mei 2016
C1 protocol versie 1.3 d.d. 10 augustus 2016 TC
C1 protocol versie 1.3 d.d. 10 augustus 2016
E1 proefpersoneninformatie ENG versie 1.3 d.d. 10 augustus 2016 TC
E1 proefpersoneninformatie ENG versie 1.3 d.d. 10 augustus 2016
E1 proefpersoneninformatie NLD versie 1.3 d.d. 10 augustus 2016 TC
E1 proefpersoneninformatie NLD versie 1.3 d.d. 10 augustus 2016
H1 CV onafh. arts van der Valk, AMC

Met vriendelijke groet,
namens de Medisch Ethische Toetsingscommissie,

Mw. mr. A. Bosma,
ambtelijk secretaris
