# Supplementary material for: Poloxamer-188 as a wetting agent for microfluidic resistive pulse sensing measurements of extracellular vesicles
Source: PLoS One. 2024 May 2;19(5):e0295849. doi: 10.1371/journal.pone.0295849 (PMC11065227; doi:10.1371/journal.pone.0295849)
Supplement: S1 Data — (ZIP) [file pone.0295849.s009.zip › supporting information/FW_ METC 2015_074.pdf]

Van: Poll, T. van der (Tom)
Aan: Winkelhuijzen, H.M.; Ursinus, J.
Onderwerp: FW: METC 2015_074
Datum: woensdag 22 mei 2019 11:10:21

Van: mecamc <mecamc@amc.uva.nl>
Verzonden: woensdag 22 mei 2019 10:20
Aan: Poll, T. van der (Tom) <t.vanderpoll@amsterdamumc.nl>
CC: Cranendonk, D.R. <d.r.cranendonk@amc.uva.nl>
Onderwerp: METC 2015_074

Onderstaande brief wordt u uitsluitend per e-mail gestuurd.

Amsterdam, 21 mei 2019 **Medisch Ethische Toetsingscommissie AMC**
ons kenmerk: 2015_074#C20191104 XT4-148
betreft: telefoon: 020 56 67389
NL52925.018.15
Blood sampling for CEMM experiments

Geachte heer Van der Poll,

Bij schrijven d.d. 6 mei 2019 ontvingen wij van u een amendement betreffende bovengenoemd project.

Het amendement betreft het toevoegen van het Laboratorium Genetische Metabole Ziekten.

Dit amendement is aan de orde geweest in de vergadering van het dagelijks bestuur van onze commissie van 15 mei 2019. Het amendement heeft geen invloed op het eerdere besluit van de commissie dat het project niet onder de reikwijdte van de WMO valt.

Wij delen u gaarne mee dit amendement ter kennisgeving te hebben aangenomen.

Betrokken documenten:
A1 aanbiedingsemail d.d. 6 mei 2019
C1 protocol versie 1.9 d.d. 18 april 2019 TC
C1 protocol versie 1.9 d.d. 18 april 2019
E1 proefpersoneninformatie NL versie 1.9 d.d. 18 april 2019
E1 proefpersoneninformatie ENG versie 1.9 d.d. 18 april 2019 TC
E1 proefpersoneninformatie ENG versie 1.9 d.d. 18 april 2019
E1 proefpersoneninformatie NL versie 1.9 d.d. 18 april 2019 TC

Met vriendelijke groet,
namens de Medisch Ethische Toetsingscommissie AMC,

Mw. mr. A. Bosma,
ambtelijk secretaris

c.c. D.R.Cranendonk
