# Supplementary material for: Poloxamer-188 as a wetting agent for microfluidic resistive pulse sensing measurements of extracellular vesicles
Source: PLoS One. 2024 May 2;19(5):e0295849. doi: 10.1371/journal.pone.0295849 (PMC11065227; doi:10.1371/journal.pone.0295849)
Supplement: S1 Data — (ZIP) [file pone.0295849.s009.zip › supporting information/O-20040430ori01_A1 C20161751 d.d. 6 september 2016-nl-en-US.pdf]

P.L.M. Walta
From: mecamc
Sent: Tuesday, September 6, 2016 16:45
To: T. van der Poll
Subject: MEC 2015_074

The following letter is sent to you by email only.

Amsterdam, September 6, 2016
our ref: 2015_074#C20161751
subject:

Medical Ethics Committee
E2-172
telephone: +31 (0)20 56 67389

NL52925.018.15
Blood sampling for CEMM experiments

Dear Mr. Van der Poll:

We received an email dated August 17, 2016 from Duncan Kranendonk containing the revised protocol for the above-mentioned study. This was discussed in our Committee meeting of September 1, 2016.
During the discussion the following issue arose:

We already indicated in our letter dated April 14, 2015 that the project is not subject to the requirements of the Medical Research (Human Subjects) Act provided certain conditions are met. It is the opinion of the Committee that the protocol and the information for study subjects are now set up in such a way as to meet the conditions for the project to be exempt from the requirements of the Medical Research (Human Subjects) Act.

We are pleased to inform you that we have received these documents for information purposes.

Relevant documents:
A1 submission letter dated March 16, 2015
A1 submission email dated March 19, 2015
A1 submission email dated January 20, 2016
A1 submission email dated May 31, 2016
A1 submission email dated June 1, 2016
A1 submission email dated August 17, 2016
B2 AMC appendix signed and dated May 25, 2016
C1 protocol version 1.3 dated August 10, 2016 TC
C1 protocol version 1.3 dated August 10, 2016
E1 subject information ENG version 1.3 dated August 10, 2016 TC
E1 subject information ENG version 1.3 dated August 10, 2016
E1 subject information NLD version 1.3 dated August 10, 2016 TC
E1 subject information NLD version 1.3 dated August 10, 2016
H1 resume of independent physician Van der Valk, AMC

Kind regards,
On behalf of the Medical Ethics Committee,

Ms. A. Bosma LLM,
Administrative Secretary

S. M. Hunter-Human BTr
Beëdigd vertaler - Wbtv nr. 5177

P.L.M. Walta

Van: mecamc
Verzonden: dinsdag 6 september 2016 16:45
Aan: T. van der Poll
Onderwerp: MEC 2015_074

Onderstaande brief wordt u uitsluitend per email gestuurd.

Amsterdam, 6 september 2016
ons kenmerk: 2015_074#C20161751
betreft:
NL52925.018.15
Blood sampling for CEMM experiments

Medisch Ethische Toetsingscommissie
E2-172
telefoon: 020 56 67389

Geachte heer Van der Poll,

Per email d.d. 17 augustus 2016 ontvingen wij van Duncan Kranendonk het herziene protocol betreffende bovengenoemde studie. Dit is besproken in de vergadering van onze commissie van 1 september jongstleden. Daarbij kwam het volgende aan de orde:

In onze brief van 14 april 2015 hebben wij al aangegeven dat het project onder voorwaarden niet WMO-plichtig is.
De commissie is van oordeel dat het protocol en de proefpersoneninformatie nu dusdanig zijn opgezet dat aan de voorwaarden voor niet WMO-plichtigheid voor dit project is voldaan.

Wij delen u gaarne mee deze documenten ter kennisgeving te hebben aangenomen.

Betrokken documenten:
A1 aanbiedingsbrief d.d. 16 maart 2015
A1 aanbiedingsemail d.d. 19 maart 2015
A1 aanbiedingsemail d.d. 20 januari 2016
A1 aanbiedingsemail d.d. 31 mei 2016
A1 aanbiedingsemail d.d. 1 juni 2016
A1 aanbiedingsemail d.d. 17 augustus 2016
B2 AMC appendix getekend d.d. 25 mei 2016
C1 protocol versie 1.3 d.d. 10 augustus 2016 TC
C1 protocol versie 1.3 d.d. 10 augustus 2016
E1 proefpersoneninformatie ENG versie 1.3 d.d. 10 augustus 2016 TC
E1 proefpersoneninformatie ENG versie 1.3 d.d. 10 augustus 2016
E1 proefpersoneninformatie NLD versie 1.3 d.d. 10 augustus 2016 TC
E1 proefpersoneninformatie NLD versie 1.3 d.d. 10 augustus 2016
H1 CV onafh. arts van der Valk, AMC

Met vriendelijke groet,
namens de Medisch Ethische Toetsingscommissie,

Mw. mr. A. Bosma,
ambtelijk secretaris

S. M. Hunter-Human BTr
Beëdigd vertaler nr. 5177

I, Susanna Margaretha Hunter-Human, registered as a sworn translator for the English and Dutch languages by the District Court of The Hague, hereby certify that the above is a true and accurate English translation of the attached Dutch document

Susanna Margaretha Hunter-Human

Amsterdam, May 12, 2020

Sworn translator for the Dutch and English languages,
registered in the Dutch Register of Sworn Interpreters and Interpreters (www.bureauwbtv.nl)
under Number 5177
