# Supplementary material for: Poloxamer-188 as a wetting agent for microfluidic resistive pulse sensing measurements of extracellular vesicles
Source: PLoS One. 2024 May 2;19(5):e0295849. doi: 10.1371/journal.pone.0295849 (PMC11065227; doi:10.1371/journal.pone.0295849)
Supplement: Attachment — Submitted filename: Reviewer_comments.pdf [file pone.0295849.s010.pdf]

**Poloxamer-188 as a wetting agent for microfluidic resistive pulse sensing measurements of extracellular vesicles**

The study effectively demonstrates the viability of employing Polaxamer-188 as a substitute for the utilization of BSA or Tween20 as wetting agents in EV determinations via MRPS. I would like to outline certain recommendations to be integrated into the manuscript prior to its publication:

<u>EV test sample characterization by FCM.</u>

The authors employ FCM as a technique for the characterization of EVs, given that it does not require the use of wetting agents and is therefore suitable for assessing the effect of these agents on EVs. As they appropriately clarify, the size detection limit of this technique is approximately 145-165 nm. However, in the Results section, they mention that almost all of the particles in the sample are EVs, when they are only observing those larger than 165 nm using this technique. In fact, in Figure 2A, for particles smaller than 280 nm, there appears to be a decrease in the level of CD235a labeling. While this is more clearly explained in the discussion, it would be useful to mention it in the Results section. It is not evident from this figure whether the data points correspond to a single experiment or are the result of experimental replicates. The same Figure illustrates the effect of Triton X-100 lysis on the particles, indicating that 98% of them were susceptible. However, this seems to hold true for particles larger than 400 nm, but not for the smaller ones. I suggest clarifying how the percentage of lysis is determined in this section.

<u>Effect of wetting agents on EV samples determined by FCM.</u>

In Figure 3, the effect of Tween20 on the particles is evident, but it is not specified whether the results are experimental replicates or single measurements.

<u>Effect of Poloxamer-188 on MRPS determination.</u>

The authors demonstrate a narrower distribution of transit time values when using polaxomer-188 compared to BSA or Tween20, indicating a higher degree of pore wetting (Figure 4). It would be useful to include a measure of dispersion for the values obtained in both cases as supplementary information.

Additionally, I suggest presenting the EV concentration values obtained with Tween20 in parallel, in order to showcase the impact of the wetting agent on the final EV concentration determined by MRPS, according to the agent used. This presentation could also reveal whether there is an increase in the concentration of EVs smaller than 200 nm that corresponds to the findings suggested in the FCM assays. Such findings would support the hypothesis of lysis or micelle formation of Tween20, detectable within this size range.

Minor Observations

Figure 1A C: The figure caption mentions that the graph corresponds to Run 2, while the text states that it corresponds to Run 1.

Figures S1.1 and S2.1: Please label panels A and B for clarity.
