# Supplementary material for: Poloxamer-188 as a wetting agent for microfluidic resistive pulse sensing measurements of extracellular vesicles
Source: PLoS One. 2024 May 2;19(5):e0295849. doi: 10.1371/journal.pone.0295849 (PMC11065227; doi:10.1371/journal.pone.0295849)
Supplement: Attachment — Submitted filename: Response to Editor.docx [file pone.0295849.s011.docx]

Editor:

1. Please note that funding information should not appear in the Acknowledgments section/Funding section or Any other areas of your Manuscript. We will only publish funding information present in the Funding Statement section of the online submission form. Please remove any funding-related text from the manuscript.

Response: We would like to express our gratitude to the editor for reviewing our manuscript. After carefully considering the provided comments, we made the necessary adjustments, including the removal of the Funding section from the revised manuscript.

Editor:

2. We are unable to open your Supporting Information file [ supporting information.7z ]. Please kindly revise as necessary and re-upload.

Response: We have uploaded the Supporting Information file in a new compressed format.

Editor:

3. Thank you for updating your data availability statement. You note that your data are available within the Supporting Information files, but no such files have been included with your submission. At this time we ask that you please upload your minimal data set as a Supporting Information file, or to a public repository such as Figshare or Dryad.

Please also ensure that when you upload your file you include separate captions for your supplementary files at the end of your manuscript.

As soon as you confirm the location of the data underlying your findings, we will be able to proceed with the review of your submission.

Response:

- Thank you for bringing this to our attention. We acknowledge that the data availability statement has been updated. We now state: "All relevant data can be found in the following Figshare repositories. Flow cytometry data can be found at: <https://doi.org/10.6084/m9.figshare.23260886>, and <https://doi.org/10.6084/m9.figshare.23261090>. Microfluidics resistive pulse sensing data can be found at: <https://doi.org/10.6084/m9.figshare.23559945>. The minimal data set underlying the results can be found at <https://doi.org/10.6084/m9.figshare.24298507>"
- Could you please provide guidance on how I can address "include separate captions for your supplementary files at the end of your manuscript" and ensure compliance with your request? I initially added the Figure and table captions of the supplementary files to the end of the manuscript based on the guideline of the PLOS ONE journal.

Editor:

4. Please amend your current ethics statement to include the full name of the ethics committee/institutional review board(s) that approved your specific study.

Response: In the "Ethics Statement" field of the submission form, we included the following information: "The Amsterdam UMC Ethical Review Board, location AMC , has stated that collecting blood from healthy donors for preclinical research is exempt from the WMO (the Medical Research Involving Human Subject Act). Hence, no additional approval from the board was required. All procedures involving human participants adhered to the Declaration of Helsinki, and verbal informed consent was obtained." Additionally, we have incorporated the same statement into page 6 of the revised manuscript, as follows: "The Amsterdam UMC Ethical Review Board, location AMC , has stated that collecting blood from healthy donors for preclinical research is exempt from the WMO (the Medical Research Involving Human Subject Act). Consequently, no additional approval from the board was required. All procedures involving human participants adhered to the Declaration of Helsinki, and verbal informed consent was obtained. "

Editor:
5.  In the Methods section please include the informed consent statement to reflect whether "written or verbal" informed consent was obtained from all participants for inclusion in the study.
Once you have amended this/these statement(s) in the Methods section of the manuscript, please add the same text to the “Ethics Statement” field of the submission form (via “Edit Submission”).

Response: We explained the informed consent statement in page 7 of the original manuscript "All procedures involving human participants adhered to the Declaration of Helsinki, and **verbal informed consent** was obtained. "
